# Automated assessment of small bowel and colon cleansing in enteroscopy using a convolutional neural network

**DOI:** 10.1055/a-2778-5666

**Published:** 2026-01-19

**Authors:** Pedro Marílio Cardoso, Miguel Mascarenhas, Miguel Martins, Francisco Mendes, João Afonso, Tiago Ribeiro, Maria João Almeida, Joana Mota, Patrícia Andrade, Helder Cardoso, João Ferreira, Guilherme Macedo

**Affiliations:** 1 285211 Gastroenterology, Centro Hospitalar Universitário de São João, Porto, Portugal; 2 26706 Faculty of Medicine, University of Porto, Porto, Portugal; 3 112048 Department of Mechanical Engineering, University of Porto, Faculty of Engineering, Porto, Portugal

**Keywords:** Endoscopy Lower GI Tract, Endoscopy Small Bowel, Quality and logistical aspects, Preparation

## Abstract

**Background and study aims:**

Device-assisted enteroscopy (DAE) offers a comprehensive examination of the gastrointestinal tract, yet its diagnostic and therapeutic success is dependent on adequate bowel preparation. Current methods for assessing preparation quality are subjective and limited to specific gastrointestinal segments. Although prior research explored artificial intelligence models for colon preparation classification, this study aimed to develop a convolutional neural network (CNN) for automatic evaluation of bowel cleanliness in DAE, addressing both small bowel and colon cleansing.

**Patients and methods:**

We retrospectively analyzed 28 procedures (single balloon, double-balloon, and motorized spiral enteroscopy from January 2023 to May 2024). Bowel preparation was graded as excellent (≥ 90% visible mucosa), satisfactory (50%-90%), or unsatisfactory (< 50%). A dataset of 88,623 images (training: 90%, testing: 10%) was used, covering both small bowel and colon areas. CNN performance was evaluated against expert consensus using sensitivity, specificity, accuracy, and area under a receiver operating characteristic (AUC-ROC).

**Results:**

The CNN demonstrated the following performance metrics: excellent cleansing (sensitivity: 97.8%, specificity: 80.3%, accuracy: 90.6%, AUC-ROC: 0.95), satisfactory cleansing (sensitivity: 81.8%, specificity: 97.9%, accuracy: 92.7%, AUC-ROC: 0.95), and unsatisfactory cleansing (sensitivity: 68.7%, specificity: 99.5%, accuracy: 96.8%, AUC-ROC: 0.96).

**Conclusions:**

Current bowel cleanliness assessment methods are subjective and region-specific. This study presents the first CNN capable of panendoscopic bowel cleanliness evaluation during DAE, achieving high accuracy and demonstrating potential for real-time clinical application. This study marks a key step toward standardizing cleanliness assessment and endoscopy quality improvement.

## Introduction

Device-assisted enteroscopy (DAE) is an endoscopic procedure that combines diagnostic capabilities with tissue sampling and therapeutic interventions. Originally designed for investigation of small bowel (SB) pathology, DAE has proven its utility across the entire gastrointestinal tract, making it a valuable tool for a range of clinical applications 1 2 . DAE comprises single and double-balloon enteroscopy, plus motorized spiral enteroscopy (now discontinued). In clinical practice, capsule endoscopy (CE) is widely regarded as the first-line investigation for SB disorders due to its noninvasive nature and comprehensive visual coverage 1 . DAE typically plays a complementary role, particularly in evaluation of SB lesions identified by CE. Ulcers and erosions are common findings, often linked to conditions like Crohn’s disease, refractory celiac disease, and infections 3 4 . DAE enhances diagnostic accuracy for small bowel Crohn’s disease by examining a greater length of ileal mucosa than conventional ileocolonoscopy and allows therapeutic interventions like balloon dilation for strictures 5 . It is also crucial for managing SB tumors identified in CE, providing tissue sampling and lesion marking. It also plays an important role in polyposis syndromes, allowing endoscopic polypectomy. In addition, DAE is commonly used in obscure gastrointestinal bleeding, especially after positive CE findings, enabling interventions like argon plasma coagulation for angioectasias 6 . DAE is also useful in technically challenging situations, providing higher cecal intubation rates and reduced discomfort in patients with difficult or incomplete colonoscopies 7 . These alternative applications highlight the need to enhance DAE diagnostic accuracy, not only for SB evaluations but also in a panendoscopic setting to maximize its clinical utility.

In both CE and DAE, quality of mucosal visualization—and consequently, the diagnostic and therapeutic potential—depends heavily on cleanliness of the gastrointestinal tract, which can be compromised by air bubbles, bile, or intestinal debris 8 9 . SB cleanliness in CE can be assessed using various qualitative and semiquantitative scales, although these methods differ in technical features and reproducibility. Currently, this evaluation relies on both operator-dependent scoring systems, such as Brotz and Eliakim score, and automated methods 10 11 12 . Automated scores are recognized for their objectivity, reliability, and reproducibility, effectively overcoming limitations associated with operator-dependent approaches 13 . However, no published or validated scales currently exist for evaluating SB cleanliness in DAE.

As previously stated, enteroscopy can also be used for diagnostic and therapeutic procedures in other parts of the gastrointestinal tract. For instance, in the colon, validated scales are available; however, these are specific to the colon and do not apply to other regions. Several assessment tools, including the Aronchick Scale 14 , the Ottawa Bowel Preparation Scale 15 , and the Boston Bowel Preparation Scale (BBPS) 16 , have been developed. However, these methods also have certain limitations because subjective evaluations by endoscopists can lead to variability in interobserver assessments, and scoring bowel preparation status after the procedure may be less accurate due to dependence on endoscopist recall 17 .

The value and reliability of DAE procedure is dependent on bowel cleanliness achieved, which, if inadequate, undermines reliability of failure to detect abnormalities. Thus, it is essential to have a reliable, objective, and reproducible scoring tool to assess the quality of SB and colon preparation in DAE. This need has prompted development of artificial intelligence (AI) algorithms to automatically assess SB 18 and colon 19 cleanliness in CE exams. Recent developments in AI, particularly through convolutional neural networks (CNNs), have significantly impacted medicine, especially in endoscopic imaging 20 21 22 . CNNs, human cortex-inspired multilayer architecture networks, have high proficiency in image pattern recognition and detection and have been developed for several purposes in CE and DAE. In fact, our group has tested AI application in DAE for identification of vascular lesions 23 , protuberant lesions 24 , ulcers, and erosions 25 and even development of a CNN capable of multi-lesion detection 26 .

Nevertheless, implementation of AI models for DAE is still in the early stages and although AI can enhance lesion detection, a clean enteric and colonic mucosa remains an essential aspect. Despite the significant potential of CNNs to automatically and objectively evaluate bowel preparation status during endoscopy procedures, research on AI models for assessing colon cleanliness during colonoscopy is limited, often restricted to single-center and controlled conditions 27 28 29 30 31 . Indeed, AI application in assessing bowel preparation quality in DAE is still underexplored. To fill this gap, our study developed and validated a CNN-based algorithm for automatic evaluation of bowel preparation quality, using a large, real-world dataset of DAE images.

## Methods

### Study design

A total of 28 DAE exams performed at São João University Hospital, between January 2023 and May 2024 were used for development of the CNN. During that period, DAE was performed by experienced gastroenterologists using three different devices: the double-balloon enteroscopy system Fujifilm EN-580T (n = 18), the single-balloon enteroscopy system Olympus EVIS EXERA II SIF-Q180 (n = 8) and the Olympus PowerSpiral Motorized Enteroscope PSF-1 (n = 2). The complete video of the examinations performed was reviewed, extracting 88,623 images in total. The images were continuously extracted by decomposing the video into single frames using a video decomposition program.

This study respected the Declaration of Helsinki and was developed in a non-interventional fashion. The study was approved by the Ethics Committee of São João University Hospital/Faculty of Medicine of the University of Porto (No. CE 407/2020). Omission of potentially identifying information of the subjects was ensured and each patient received a random number assignment to obtain effective data anonymization for researchers involved in the CNN. A legal team with Data Protection Officer certification was responsible for non-traceability of the data in conformity with general data protection regulation.

### Classification of bowel preparation

**Fig. 1:**
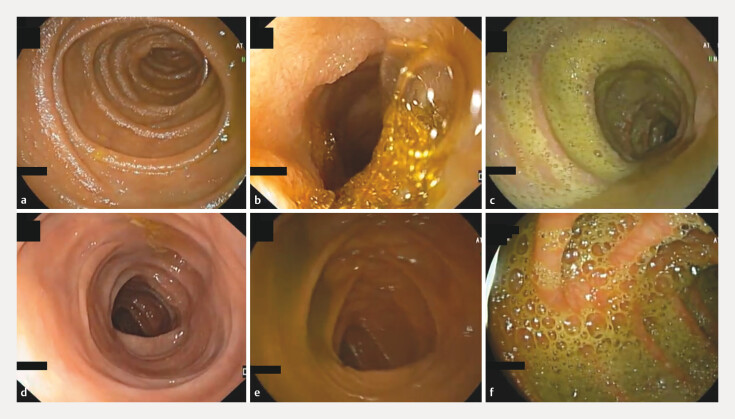
Images depicting quality of bowel preparation. **a** SB – excellent. **b** SB – satisfactory. **c** SB – unsatisfactory. **d** C – excellent. **e** C – satisfactory. **f** C - unsatisfactory. C, colon; SB, small bowel.

An experienced gastroenterologist analyzed the still frames independently and scored the quality of SB and colon cleansing based on the proportion of mucosa visualized and in accordance with the degree of obscuration by bubbles, bile and/or debris. Images were divided into three groups accordingly to quality of cleansing in each still frame. They were categorized as excellent (E) when ≥ 90% of the mucosa was visible, satisfactory (S) when 50% to 90% of mucosa was visible, and unsatisfactory (U) when < 50% of the mucosa was visible ( Fig. 1 ). This classification was independent of presence or absence of any endoscopic lesions. The final classification of each frame required a consensus between three experienced gastroenterologists. When a common agreement was not possible, the frame was excluded.

### CNN development

**Fig. 2:**
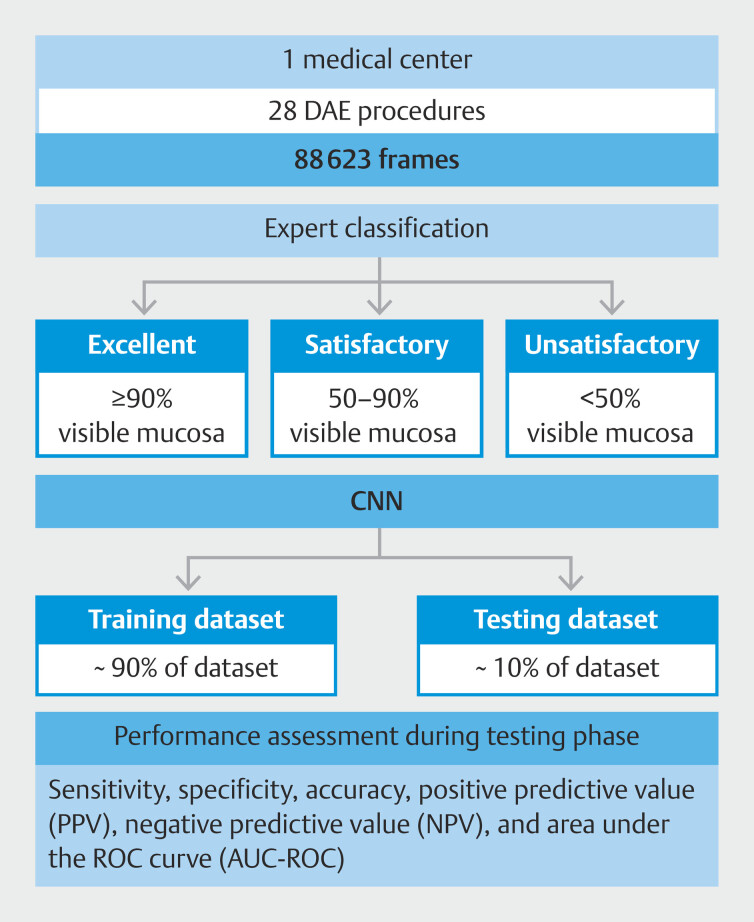
Flowchart of procedures carried out in the training and testing phases of the study.

A CNN was designed to provide automatic classification of SB and colon preparation according to the aforementioned categories, enabling a panendoscopic

cleaning assessment. A total of 88,623 images were included and the total dataset was divided into training and testing sets using a patient-split approach, ensuring that data from the same patient did not appear in both sets simultaneously. From the complete data set, 90% (n = 79761) was used to develop and train the algorithm. The remaining 10% (n = 8862) was used to validate CNN performance independently. Fig. 2 represents a graphical flowchart of the study design and CNN development.

The CNN was created with the Xception model pre-trained on ImageNet. The convolutional layers of the model were kept, assuring transference of the learning to our data, whereas the last fully connected layers were removed. Attachment of fully connected layers was based on the number of the classes for classification of DAE images. The model had two blocks with fully connected layers followed by a Dropout layer of 0.25 drop rate. A Dense layer with a size based on the number of categories to classify was added. A learning rate of 0.0001, batch size of 64, and number of epochs of 20 was set by trial and error. Our group used Keras libraries and Tensor-flow 2.3 to prepare the data and run the model. The analysis was dependent on a computer with an Intel Xeon Gold 6130 processor (Intel, Santa Clara, California, United States) and a NVIDIA Quadro RTXTM 4000 graphic processing unit (NVIDIA Corporate, Santa Clara, California, United States).

### Model performance and statistical analysis

**Fig. 3:**
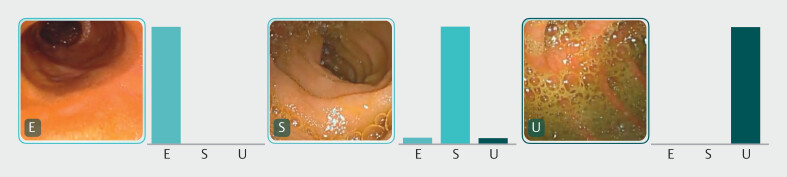
Obtained output of CNN implementation (bars represent the probability estimated by the network and the finding with the highest probability was considered the predicted classification output). E, excellent bowel preparation; S, satisfactory bowel preparation; U, unsatisfactory bowel preparation.

The trinary CNN calculated the probability of each category in each image. The probability that the trained CNN would attribute each of the three categories to an image (excellent, satisfactory, or unsatisfactory) was estimated, with higher probabilities demonstrating greater CNN prediction confidence, such that the category carrying the highest probability score was considered as the classification output predicted by the CNN ( Fig. 3 ).

The main outcome measures encompassed sensitivity and specificity to distinguish the three cleanliness categories, along with positive predictive value (PPV), negative predictive value (NPV), and overall accuracy. Moreover, receiver operating characteristic curves (ROCs) and area under the curve (AUC) were used to assess performance of the CNN to detect and differentiate the different SB and colon preparation classes.

Furthermore, CNN image processing efficiency was assessed by measuring the time it took for the CNN to classify all validation images in the test dataset. Statistical analyses were carried out with Sci-Kit learn v.22.2 software 32

## Results

### CNN construction and training

Based on 28 DAE procedures performed, the CNN model was constructed with 88,623 images. Of the 28 procedures undertaken, 18 were carried out using double-balloon enteroscope (n = 56 972 images), eight using the single-balloon enteroscope (n = 25,321 images), and two using the PowerSpiral enteroscope (n = 6330 images).

From this cohort of examinations, in the training phase, 9351 were labeled by the experts as an excellent preparation, 5187 were labeled as satisfactory preparation, and 1414 were labeled as unsatisfactory preparation. The training and validation datasets were built for the design of the CNN incorporating 90% (n = 79761) and 10% (n = 8862) respectively.

Because the data were repeatedly used as inputs to the multilayer CNN, overall accuracy of the network was not only enhanced in the training period but also in the validation environments, reflecting the ability of the CNN to learn.

### CNN global performance in differentiating classification of bowel cleanliness during testing

**Fig. 4:**
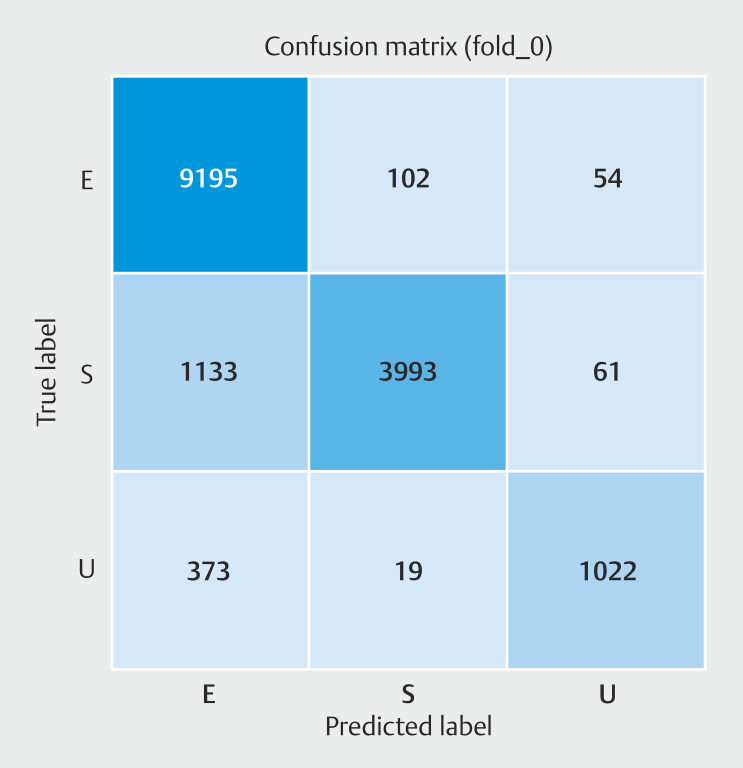
Confusion matrix of the CNN automatic prediction of bowel cleanliness versus expert classification (true label).

CNN performance was evaluated using an independent dataset of images ( Fig. 4 ). Performance was assessed based on sensitivity, specificity, PPV, NPV, accuracy, and AUC. Overall, the DAE deep learning algorithm proved to be capable of automatically differentiating bowel preparation classes with a calculated accuracy of 89.1%, sensitivity of 87.6%, and specificity of 92.2%.

**Fig. 5:**
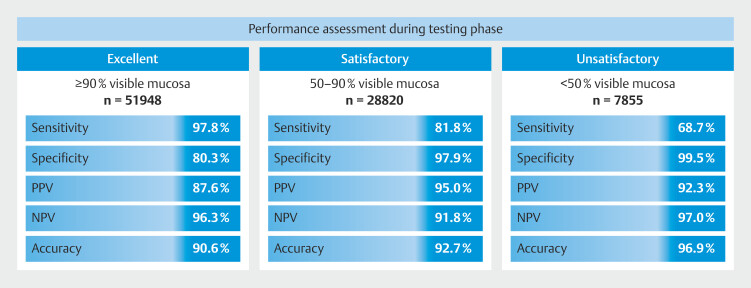
Performance assessment during testing phase for each cleanliness classification.

During the testing stage, for the category of excellent preparation, the model achieved a mean sensitivity of 97.8%, specificity of 80.3% and overall accuracy of 90.6%. For the satisfactory category, the model had sensitivity of 81.8%, specificity of 97.9%, and overall accuracy of 92.7%. For images of unsatisfactory preparation, the model achieved a mean sensitivity of 68.7%, specificity of 99.5%, and overall accuracy of 96.9%. The CNN completed evaluation with an image processing time of 170 images per second. Individual performance metrics for each of the categories are shown in Fig. 5 .

**Fig. 6:**
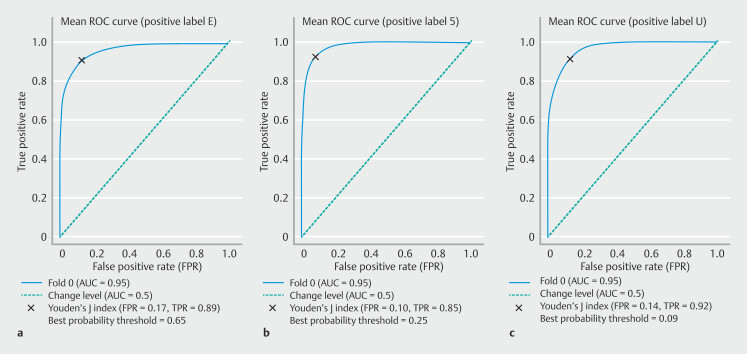
Receiver operating characteristic (ROC) curve of CNN performance in differentiating the SB and colon preparation classes for each prediction. **a** Excellent. **b** Satisfactory. **c** Unsatisfactory.

ROC analyses and respective AUCs ( Fig. 6 ) indicated that performance of the CNN in differentiating excellent, satisfactory, and unsatisfactory cleanliness in SB and colon preparations were high, with AUCs of 0.95, 0.95 and 0.96, respectively.

## Discussion

Despite the importance of bowel cleanliness, current assessment methods remain subjective and limited to specific segments of the gastrointestinal tract, such as BBPS for colonoscopy. Although it remains the most validated scale, it has notable limitations in clinical practice, including need for segmental scoring, variability in reporting, and potential for inaccuracies when scores are assigned retrospectively 33 . Although several qualitative and semiquantitative scales exist to assess SB cleanliness in CE, these vary in technical characteristics and reproducibility 13 . To date, no equivalent tools exist for evaluating SB and colon cleanliness.

AI has emerged as a transformative tool in healthcare, with some of its most interesting and promising applications in gastrointestinal endoscopy 21 22 34 . Algorithms for polyp detection and characterization have already entered clinical practice, reflecting the urgency to address quality standards in endoscopy and mitigate operator dependency 6 . This rapid translation underscores the potential of AI to enhance performance measures and quality metrics, such as bowel preparation adequacy. Suboptimal preparation is associated with missed polyps, incomplete procedures, increased healthcare costs, and reduced patient satisfaction 33 . Addressing these challenges requires innovative solutions, and AI offers a promising path forward.

This study demonstrates the feasibility and efficacy of a CNN-based approach for objectively assessing bowel cleanliness during DAE. The proposed neural network achieved high performance metrics, with overall accuracies exceeding 90% for excellent, satisfactory, and unsatisfactory bowel preparation. DAE is an endoscopic modality with both diagnostic and therapeutic purposes focused on both SB and colon pathology and, therefore, evaluation of bowel preparation is essential. The ability to classify bowel preparation ensures that quality of visualization, a critical factor for detecting lesions and conducting therapeutic interventions, is objectively assessed.

Our literature review identified previous efforts in AI use to assess bowel preparation in colonoscopy. In 2020, Su et al. develop a deep learning-based automatic quality control system that considered withdrawal time and scope stability, bowel preparation, and polyp detection 27 . In 2020, a paper published by Zhou et al. described a neural network capable of providing bowel preparation scores during endoscope withdrawal 28 . In 2022, a group from Canada used 28 colonoscopy videos and developed two CNNs to determine BBPS subclassification and bowel preparation adequacy with overall good performance metrics 29 . Last year, Lee et al. published an evaluation of the clinical applicability of an AI model for bowel preparation evaluation 31 .

Prior studies on AI-driven cleanliness evaluation in CE, such as those by Ribeiro et al. and Mascarenhas Saraiva et al., also demonstrated feasibility of CNNs in automated preparation assessment in that context 18 19 .

Our performance metrics are consistent with prior studies exploring the potential of deep learning tools for bowel preparation assessment, but unlike previous approaches, this study presents the first CNN capable of automatic classification of bowel cleansing quality during DAE, encompassing both the SB and colon, and, therefore, being a step closer to a panendoscopic assessment.

The CNN was trained and evaluated with data from several enteroscopes such as single-balloon, double-balloon, and motorized spiral enteroscope. This multidevice approach ensures interoperability and enhances model generalizability across multiple technologies, broadening its clinical applicability.

The robust performance across all categories highlights the CNN capacity to handle variability in imaging conditions and anatomical segments. The model ability to process images at a high rate demonstrates its suitability for real-time clinical application.

By integrating these capabilities, this study provides a novel framework for improving diagnostic and therapeutic outcomes in gastrointestinal endoscopy. The implications of panendoscopic automated cleanliness evaluation are significant. By delivering an objective and reproducible evaluation, AI systems have the potential to enhance the quality of endoscopy, minimize missed lesions, and standardize quality criteria across institutions.

Despite these advances, the study has several limitations. The dataset was derived from a single center, which may limit external validity. Although the multidevice design enhances generalizability, multicenter studies are necessary to confirm CNN applicability across broader patient populations and clinical settings. In addition, the performance for unsatisfactory cleansing, although reasonable, showed lower sensitivity, suggesting room for improvement in detecting severely compromised preparation. This is crucial, because inadequate preparation is associated with increased rates of missed lesions and post-colonoscopy colorectal cancer. Another limitation is that the CNN was trained and validated using still images, which do not capture the dynamic nature of DAE procedures. Adapting the model to analyze real-time video streams will be essential for clinical application. In addition, the study did not assess how variations in bowel preparation protocols influenced CNN performance. Evaluating the model across different preparation protocols could provide insights for optimizing bowel cleanliness in a patient-personalized approach. Expanding the CNN to include both cleanliness assessment and lesion detection in a single tool would further enhance its utility, allowing endoscopists to identify clinically significant findings more effectively. Ethical and legal considerations, such as establishing clear accountability protocols for AI decision-making and ensuring clinician-AI collaboration, are also critical for safe implementation.

The future of endoscopy is shifting toward a panendoscopic approach, in which CE serves as the primary diagnostic tool, and DAE might act as its therapeutic extension. Deep learning assessment tools will play a key role in objectively evaluating bowel cleanliness, preventing misinterpretation due to inadequate preparation. In addition, understanding how bowel preparation affects AI performance will be essential to refine algorithms and improve their clinical reliability.

## Conclusions

This study represents a pivotal step in development of deep learning algorithms for panendoscopic bowel preparation evaluation during DAE. Future research will focus on developing the model for real-time integration, validation in larger multicenter studies, and exploration of its integration into other existing endoscopy platforms. In conclusion, incorporating advanced AI techniques into panendoscopic procedures offers significant potential to standardize cleanliness assessment, enhance diagnostic accuracy, and improve the quality and accountability of gastrointestinal endoscopy that will ultimately improve patient outcomes.

## References

[1] Pennazio M Rondonotti E Despott EJ Small-bowel capsule endoscopy and device-assisted enteroscopy for diagnosis and treatment of small-bowel disorders: European Society of Gastrointestinal Endoscopy (ESGE) Guideline - Update 2022 Endoscopy 2023 55 58 95 10.1055/a-1973-3796 36423618

[2] Rondonotti E Spada C Adler S Small-bowel capsule endoscopy and device-assisted enteroscopy for diagnosis and treatment of small-bowel disorders: European Society of Gastrointestinal Endoscopy (ESGE) Technical Review Endoscopy 2018 50 423 446 10.1055/a-0576-0566 29539652

[3] Yen HH Chang CW Chou JW Balloon-assisted enteroscopy and capsule endoscopy in suspected small bowel Crohn's disease Clin Endosc 2017 50 417 423 10.5946/ce.2017.142 29017295 PMC5642058

[4] Rondonotti E Koulaouzidis A Yung DE Neoplastic Diseases of the Small Bowel Gastrointest Endosc Clin North Am 2017 27 93 112 10.1016/j.giec.2016.08.005 27908521

[5] Bettenworth D Bokemeyer A Kou L Systematic review with meta-analysis: efficacy of balloon-assisted enteroscopy for dilation of small bowel Crohn's disease strictures Aliment Pharmacol Therap 2020 52 1104 1116 32813282 10.1111/apt.16049 PMC8052861

[6] Messmann H Bisschops R Antonelli G Expected value of artificial intelligence in gastrointestinal endoscopy: European Society of Gastrointestinal Endoscopy (ESGE) Position Statement Endoscopy 2022 54 1211 1231 10.1055/a-1950-5694 36270318

[7] Despott EJ Murino A Nakamura M A prospective randomised study comparing double-balloon colonoscopy and conventional colonoscopy in pre-defined technically difficult cases Digest Liver Dis 2017 49 507 513 10.1016/j.dld.2017.01.139 28314604

[8] Belsey J Crosta C Epstein O Meta-analysis: efficacy of small bowel preparation for small bowel video capsule endoscopy Curr Med Res Opin 2012 28 1883 1890 10.1185/03007995.2012.747953 23136911

[9] Viazis N Sgouros S Papaxoinis K Bowel preparation increases the diagnostic yield of capsule endoscopy: a prospective, randomized, controlled study Gastrointest Endosc 2004 60 534 538 10.1016/s0016-5107(04)01879-6 15472674

[10] Ponte A Pinho R Rodrigues A Review of small-bowel cleansing scales in capsule endoscopy: A panoply of choices World J Gastrointest Endosc 2016 8 600 609 10.4253/wjge.v8.i17.600 27668070 PMC5027030

[11] Brotz C Nandi N Conn M A validation study of 3 grading systems to evaluate small-bowel cleansing for wireless capsule endoscopy: a quantitative index, a qualitative evaluation, and an overall adequacy assessment Gastrointest Endosc 2009 69 262 2.7E263 18851851 10.1016/j.gie.2008.04.016

[12] Eliakim R Yassin K Niv Y Prospective multicenter performance evaluation of the second-generation colon capsule compared with colonoscopy Endoscopy 2009 41 1026 1031 19967618 10.1055/s-0029-1215360

[13] Rosa B Margalit-Yehuda R Gatt K Scoring systems in clinical small-bowel capsule endoscopy: All you need to know! Endosc Int Open 2021 09 E802 E823 10.1055/a-1372-4051 PMC8159625 34079861

[14] Gerard DP Foster DB Raiser MW Validation of a new bowel preparation scale for measuring colon cleansing for colonoscopy: the chicago bowel preparation scale Clin Transl Gastroenterol 2013 4 e43 10.1038/ctg.2013.16 24304940 PMC3865439

[15] Rostom A Jolicoeur E Validation of a new scale for the assessment of bowel preparation quality Gastrointest Endosc 2004 59 482 486 10.1016/s0016-5107(03)02875-x 15044882

[16] Lai EJ Calderwood AH Doros G The Boston bowel preparation scale: a valid and reliable instrument for colonoscopy-oriented research Gastrointest Endosc 2009 69 620 625 10.1016/j.gie.2008.05.057 19136102 PMC2763922

[17] Parmar R Martel M Rostom A Validated scales for colon cleansing: A systematic review Am J Gastroenterol 2016 111 197 204quiz 205 26782820 10.1038/ajg.2015.417

[18] Ribeiro T Mascarenhas Saraiva MJ Afonso J Design of a convolutional neural network as a deep learning tool for the automatic classification of small-bowel cleansing in capsule endoscopy Medicina (Kaunas) 2023 59 810 37109768 10.3390/medicina59040810 PMC10145655

[19] Mascarenhas Saraiva MJ Afonso J Ribeiro T AI-driven colon cleansing evaluation in capsule endoscopy: A deep learning approach Diagnostics (Basel) 2023 13 3494 38066734 10.3390/diagnostics13233494 PMC10706297

[20] Pannala R Krishnan K Melson J Artificial intelligence in gastrointestinal endoscopy VideoGIE 2020 5 598 613 10.1016/j.vgie.2020.08.013 33319126 PMC7732722

[21] Le Berre C Sandborn WJ Aridhi S Application of artificial intelligence to gastroenterology and hepatology Gastroenterology 2020 158 76 9.4E73 31593701 10.1053/j.gastro.2019.08.058

[22] Mascarenhas M Afonso J Andrade P Artificial intelligence and capsule endoscopy: unravelling the future Ann Gastroenterol 2021 34 300 309 10.20524/aog.2021.0606 33948053 PMC8079882

[23] Mascarenhas Saraiva M Ribeiro T Afonso J Deep learning and device-assisted enteroscopy: Automatic detection of gastrointestinal angioectasia Medicina 2021 57 1378 34946323 10.3390/medicina57121378 PMC8706550

[24] Cardoso P Saraiva MM Afonso J Artificial intelligence and device-assisted enteroscopy: automatic detection of enteric protruding lesions using a convolutional neural network Clin Translat Gastroenterol 2022 13 e00514 10.14309/ctg.0000000000000514 PMC9400931 35853229

[25] Martins M Mascarenhas M Afonso J Deep-learning and device-assisted enteroscopy: Automatic panendoscopic detection of ulcers and erosions Medicina 2023 59 172 10.3390/medicina59010172 36676796 PMC9865285

[26] Mendes F Mascarenhas M Ribeiro T Artificial intelligence and panendoscopy-automatic detection of clinically relevant lesions in multibrand device-assisted enteroscopy Cancers (Basel) 2024 16 10.3390/cancers16010208 PMC10778030 38201634

[27] Su J-R Li Z Shao X-J Impact of a real-time automatic quality control system on colorectal polyp and adenoma detection: a prospective randomized controlled study (with videos) Gastrointest Endosc 2020 91 415 INF 31454493 10.1016/j.gie.2019.08.026

[28] Zhou J Wu L Wan X A novel artificial intelligence system for the assessment of bowel preparation (with video) Gastrointest Endosc 2020 91 428 INF 31783029 10.1016/j.gie.2019.11.026

[29] Low DJ Hong Z Jugnundan S Automated detection of bowel preparation scoring and adequacy with deep convolutional neural networks J Can Assoc Gastroenterol 2022 5 256 260 36467599 10.1093/jcag/gwac013 PMC9713630

[30] Haithami MS Ahmed A Liao IY Automatic bowel preparation assessment using deep learning In Lecture Notes in Computer Science 2023 574 588

[31] Lee JY Park J Lee HJ Automatic assessment of bowel preparation by an artificial intelligence model and its clinical applicability J Gastroenterol Hepatol 2024 39 1917 1923 10.1111/jgh.16618 38766682

[32] Pedregosa F Varoquaux G Gramfort A Scikit-learn: Machine learning in Python J Mach Learn Res 2011 12 2825 2830

[33] Hassan C East J Radaelli F Bowel preparation for colonoscopy: European Society of Gastrointestinal Endoscopy (ESGE) Guideline - Update 2019 Endoscopy 2019 51 775 794 10.1055/a-0959-0505 31295746

[34] Pecere S Milluzzo SM Esposito G Applications of artificial intelligence for the diagnosis of gastrointestinal diseases Diagnostics (Basel) 2021 11 10.3390/diagnostics11091575 PMC8469485 34573917

